# Understanding the Persistence of Plague Foci in Madagascar

**DOI:** 10.1371/journal.pntd.0002382

**Published:** 2013-11-07

**Authors:** Voahangy Andrianaivoarimanana, Katharina Kreppel, Nohal Elissa, Jean-Marc Duplantier, Elisabeth Carniel, Minoarisoa Rajerison, Ronan Jambou

**Affiliations:** 1 Unité Peste, Institut Pasteur de Madagascar, Antananarivo, Madagascar; 2 Unité d'Immunologie, Institut Pasteur de Madagascar, Antananarivo, Madagascar; 3 Department of Veterinary Clinical Sciences, University of Liverpool, Liverpool, United Kingdom; 4 Unité d'Entomologie, Institut Pasteur de Madagascar, Antananarivo, Madagascar; 5 IRD, CBGP (Inra/Ird/Cirad/MontpellierSupAgro), Montpellier, France; 6 Unité de Recherche Yersinia, Institut Pasteur, Paris, France; Yale University School of Medicine, United States of America

## Abstract

Plague, a zoonosis caused by *Yersinia pestis*, is still found in Africa, Asia, and the Americas. Madagascar reports almost one third of the cases worldwide. *Y. pestis* can be encountered in three very different types of foci: urban, rural, and sylvatic. Flea vector and wild rodent host population dynamics are tightly correlated with modulation of climatic conditions, an association that could be crucial for both the maintenance of foci and human plague epidemics. The black rat *Rattus rattus*, the main host of *Y. pestis* in Madagascar, is found to exhibit high resistance to plague in endemic areas, opposing the concept of high mortality rates among rats exposed to the infection. Also, endemic fleas could play an essential role in maintenance of the foci. This review discusses recent advances in the understanding of the role of these factors as well as human behavior in the persistence of plague in Madagascar.

## Introduction

Plague is a flea-borne fatal zoonosis caused by the bacillus *Yersinia pestis*. Primarily a disease of rodents and fleas, it has been responsible for three pandemics resulting in millions of deaths [1]. Despite advances in its control and understanding, plague is far from eradicated [2]. Due to its wildlife reservoirs, plague is still endemic in Asia, the Americas, and Africa. It is also reemerging in countries where the disease was thought to have disappeared [2], [3]. Civil wars, urbanization, deforestation, and mining may also have an impact on the disease.

Worldwide, bubonic plague is the predominant form and is acquired after a fleabite. The bacteria multiply at the site of inoculation and disseminate via the lymphatic system to the lymph nodes. After two to six days, a painful swelling lymph node appears (the bubo), along with high fever, headache, dizziness, and prostration. Without treatment, the infection rapidly disseminates to reach the spleen, liver, and sometimes the lungs, causing a fatal septicaemia. Without treatment, lethality occurs in 40–70% of the patients. Pneumonic plague is rare but even deadlier. It may arise from a bubonic form, by haematogenous spread to the lungs, or from inhalation of aerosols during human-to-human transmission. After one to three days of latency, the onset is sudden and always fatal without early efficient treatment. Here, we review different factors that may explain how the disease is able and continue to persist in Madagascar.

## Methods

The review of the literature was conducted using the online databases PubMed and HINARI. A thorough search was then undertaken in Madagascar from earlier works to recent findings, including dissertations and unpublished reports from the Ministry of Health and Institut Pasteur de Madagascar (which hosts the plague Malagasy reference center), with particular emphasis on plague dynamic. Altogether, documents cover almost 50 years of plague studies in Madagascar.

## Brief Overview of Plague Epidemiology

Within the *Enterobacteriaceae* family, the genus *Yersinia* includes three human pathogenic species: *Yersinia enterocolitica*, *Yersinia pseudotuberculosis*, and *Y. pestis*, the causative agent of plague [1]. Although, *Y. pestis* and *Y. pseudotuberculosis* differs radically in their virulence and transmission route, they share a high genetic homology. *Y. pestis* diverged from *Y. pseudotuberculosis* within the last 20,000 years [4].

Twenty-five hundred species and subspecies of Siphonaptera are described but only 80 of these are known to be susceptible to *Y. pestis* [5], among which the genus *Xenopsylla* (especially *Xenopsylla cheopis*) plays a major role in pandemics. Fleas of this genus are found in all domestic and peridomestic settings where humans are at risk of infection with *Y. pestis* due to its high vector efficiency and broad host preference [6]. In sub-Saharan regions and in rural areas of Brazil and India, *Xenopsylla brasiliensis* is the predominant vector for plague [6]. Other species, like *Xenopsylla astia* (Indonesia, Southeast Asia) and *Xenopsylla vexabilis* (Pacific Islands) are also important vectors [7]. The flea specificity to rodent hosts varies from one specific host to a broad affinity: in the northern United States, *Oropsylla hirsuta* parasitizes a species of prairie dogs, *Cynomys ludovicianus* [6], while in Zimbabwe, the four major rodent species *Gerbilliscus leucogaster*, *Rattus rattus*, *Rhabdomys pumilio*, and *Mastomys natalensis* are all hosts of *X. brasiliensis* [8].

The high vector efficiency of *X. cheopis* is reported to be related to its ability to get "blocked," which increases the transmission potential of *Y. pestis*. The bacterium produces biofilm required for proventricular blocking [5] of the flea leading to an increased biting rate and regurgitation of bacteria into the wound. Partial biofilm blockage is sufficient to assure transmission, as for *Oropsylla montana* (Baker) in the United States [9].

Around 200 species of rodents and lagomorphs have been connected to the epidemiology of plague so far [6], but only few are considered significant hosts [10]. Frequency of contact between human and host varies depending on the species. *R. rattus* is a tree dwelling species nesting often in the roof of huts, whereas *Rattus norvegicus* is a ground dweller, preferably living in sewer networks of large towns. *Rattus* spp. are the major reservoir of plague in parts of Asia and Africa, especially in Madagascar [3], [11], [12]. Its population dynamics determine plague dynamics [13], [14]. Other rodents are locally involved in plague epidemiology such as the great gerbil (*Rhombomys opimus*) in Kazakhstan or the black-tailed prairie dog (*C. ludovicianus*) and the ground squirrel (*Spermophilus beecheyi*) in the United States [10].

Environmental conditions modulate seasonal transmission and global distribution of plague [15], [16]. In Asia and the United States, epidemics occur at the end of winter when rodents leave their burrows after hibernation. In other foci, seasonality in the abundance of rodents is less obvious and flea dynamics seem more important to take into account. Fleas, especially immature stages, developing in host burrows are sensitive to air temperature and humidity [17] and thus are affected by soil moisture in rodent burrows. Larvae are susceptible to desiccation [15], and their survival varies inversely with air dryness. Hot and dry days also reduce blockage in fleas [17], and low temperatures delay bacterial proliferation and early-phase transmission by *X. cheopis* [18].

## Plague in Madagascar

### Plague in Madagascar: A Long History

Plague arrived in the port city of Toamasina with steamboats from India in 1898 [19]. It then spread to other harbors and reached the central highlands in 1921 following the construction of the railways. It invaded the central highlands while disappearing progressively from the coasts.

From 1957 to 2001, a total of 20,900 suspected human cases were declared with an increase in the number of districts affected. Fortunately, over the years the case fatality rate decreased from 55.7% to 20.9% [20]. Still in 2004, 1,214 cases and 98 deaths were reported, but since then the incidence of human plague cases has declined continuously. However, Madagascar still accounted for 30% of human cases worldwide from 2004 to 2009 [21].

**Figure 1:**
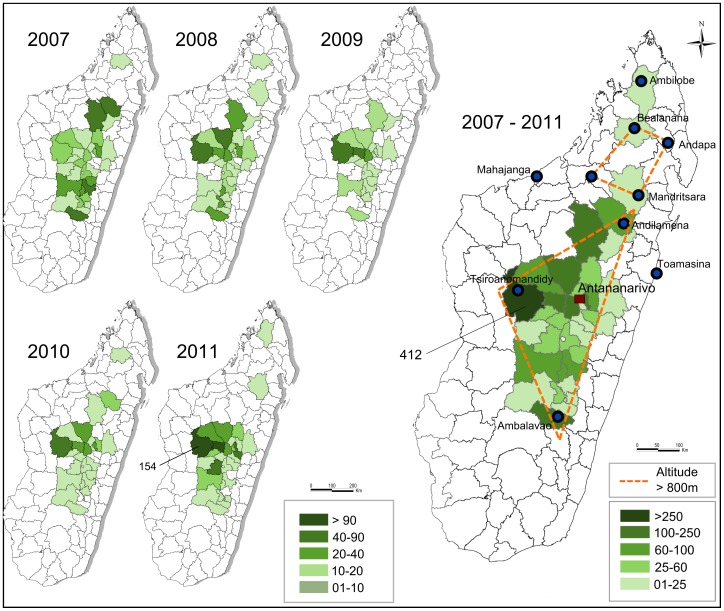
Madagascar plague mapping from 2007 to 2011. Dashed line: limits of the main plague foci (central and northern foci). Green area: districts that have notified plague cases. Most plague cases were reported from the district of Tsiroanomandidy during this period. (Sources: OCHA, Institut Pasteur de Madagascar).

Nowadays plague is endemic in rural areas of the central highlands above 800 metres of altitude. The northern plague focus is located around the Tsaratanana Mountains (Figure 1). Additionally, plague has emerged more in the north at Ambilobe in 2011 (unpublished data) between the northern foci and Antsiranana.

From 2007 to 2011, bubonic plague accounted for 86.6% of suspected cases while pneumonic and undocumented cases accounted for 9.4% and 4%, respectively. The case fatality rate was 13% for suspected cases and 18.6% for confirmed cases. Reports of pneumonic plague cases were limited to the highlands and most often evolved from bubonic plague.

### The Bacterium-Reservoir-Flea Triad in Madagascar

In Madagascar, all *Y. pestis* strains belong to the biovar Orientalis, which spread all over the world during the third pandemic. Isolates can be subdivided into four ribotypes (B, Q, R, and T), of which the most common is B, the original invading strain, while the three others are specific to Madagascar [22]. Antibiotic-resistant *Y. pestis* strains were also first isolated in Madagascar with one strain resistant to eight different antibiotics, including those used for plague prophylaxis and therapy [23].

**Figure 2:**
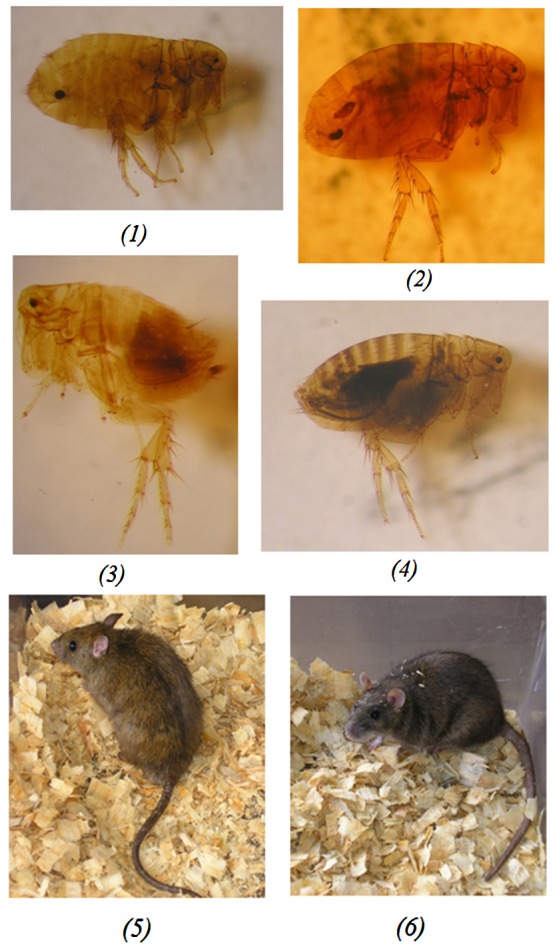
Main vectors and rodent reservoirs in Madagascar. Fleas involved in plague transmission in Madagascar: *Synopsyllus fonquerniei* female *(1)* and *Synopsyllus fonquerniei* male *(3)* are found on outdoor rats, whereas *Xenopsylla cheopis* female *(2)* and *Xenopsylla cheopis* male *(4)* live on indoor rats. Rat species involved in plague transmission in Madagascar: *Rattus rattus (5)* and *Rattus norvegicus (6)*.

Thirteen genera of Siphonaptera (four of them endemic) were described in Madagascar [24]; of those, two are involved in plague transmission: *Xenopsylla* and *Synopsyllus*. The main vector is *X. cheopis*, which parasitizes black rats living inside houses (Figure 2). The endemic genus *Synopsyllus* is composed of five species, among which *S. fonquerniei* is the most prevalent (Figure 2). It can be found in the fur or burrows of black rats living outside houses but also in open biotopes (rice fields, savannas) and in forests. This species is involved in the plague cycle above 800 metres of altitude and shows greater transmission efficiency than *X. cheopis* [25]. It also parasitizes endemic hedgehogs, rodents, and occasionally a species of lemur and insectivores.

Yet *R. rattus* remains the main plague reservoir host in Madagascar (Figure 2). Its arrival is closely linked to the history of the colonization of the island by humans [26]. The black rat is the dominant rodent species and is found everywhere: in houses, villages, fields, and also in the forests [19], [26]. Its populations can expand rapidly as it can breed inside houses all year round with an average gestation period of only 21 days and a mean litter size of 5.4 (in Madagascar). Conversely, *R. norvegicus* is limited to large towns since the 1950s, but is currently spreading on the western side of the island.

## Main Factors Impacting the Epidemiology of Plague in Madagascar

### Rural versus Urban Foci

**Figure 3:**
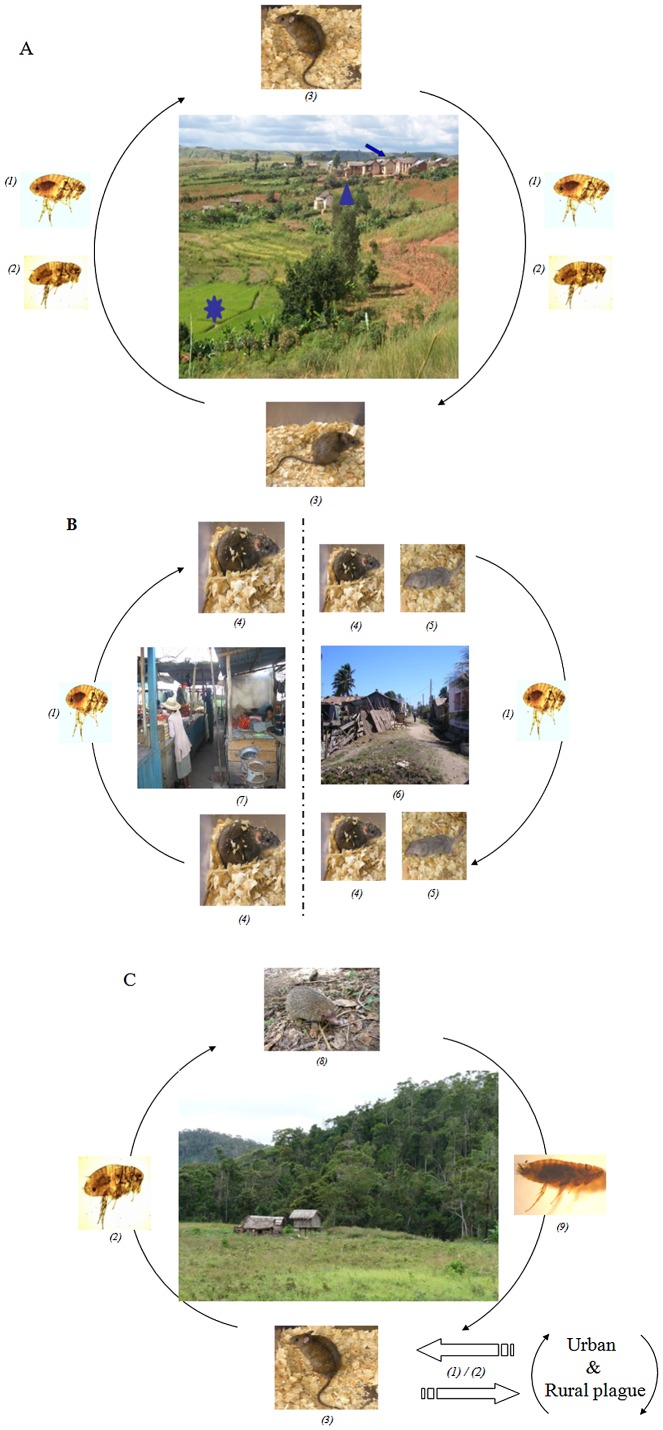
Plague transmission cycle. A) *Plague cycle in the rural area of Madagascar*. Rural plague foci of the highlands are organized into three habitats: houses (arrow), sisal hedges (arrowhead), and rice fields (star). The black rat, *R. rattus (3)*, is the main rodent involved in transmission associated with *X. cheopis (1)* and the endemic flea *S. fonquerniei (2)*. (Photo of plague foci: S. Rahelinirina). B) *Plague cycle in the urban areas of Madagascar*. Urban plague occurs mainly in the cities of Antananarivo (Isotry Market, left) *(7)* and Mahajanga (Abattoir suburb, right) *(6)*. *R. norvegicus (4)* and *X. cheopis (1)* are involved in each focus. The Asian shrew (*S. murinus*) *(5)* has long been suspected to play a major role in the epidemiological cycle of plague in Mahajanga. C) *Plague cycle in the forest area*. A sylvatic transmission occurs in Madagascar with *R. rattus (3)* and endemic micromammals (such as *Setifer setosus*) *(8)* as reservoirs. *S. fonquerniei (2)* is the major vector of the disease in this area. The role of other endemic fleas *(9)* is not yet determined. (Photo of forest of Ampahitra: S. Telfer; *Setifer setosus*: V. Soarimalala).

In Madagascar, plague is predominantly a rural disease [21] related to agricultural activities. In the highlands, there is a hot and rainy season from October to April, followed by a cold and dry season. Harvesting occurs from February to June in dry-farming areas and in May in rice fields (in some places a second rice harvesting may occur in December). Maximum abundance of rodents in the fields is observed in July and August, followed by the maximum abundance of fleas from September to November (see [7] for more details). Villages provide three distinct habitats: houses located on top of hills, sisal hedges around livestock enclosures, and irrigated rice fields in lower areas (Figure 3A). Habitat choice and population dynamics of rodents are mainly driven by the availability of resources [14]. High plague transmission to humans has been associated with low abundance of rats and an increase in flea vectors [20]. This low number of rats is due to food shortages and an interruption of reproduction of outside rat populations during the cold season [26]. Conversely during rice harvest, an increase in reproductive rate and migration from houses to sisal hedges [14] are associated with low plague transmission to humans (Table 1). These factors are impacted by climate mediated by the availability of food and shelter.

**Table 1:** Factors related to human plague.

Rural settings	High human plague season	Low human plague transmission
Period of the year	October to April	May to September
Weather	Warm and rainy	Dry and cold
Food availability	Absence of crops in the fields	Rice harvest in the fields
Rat population (*R. rattus*)	Low abundance (low reproduction/outbreaks due to plague)	High rat reproduction (inside houses)
Flea abundance	*X. cheopis* in the houses/*S. fonquerniei* outside	

Urban settings	Antananarivo city	Mahajanga coastal city
Average altitude	1,200 metres	125 metres
Period of the year	October to April	July to November
Weather	Warm and rainy	Dry and cool
Major small mammal population	*R. norvegicus* (the sewer rat)	*Suncus murinus* (the Asian shrew)
Flea abundance	*X. cheopis*	*X. cheopis*

According to [3], [14], [20], [27], [28].

Urban plague was mainly described in Mahajanga and Antananarivo (Figure 3B). The seaport of Mahajanga first experienced plague in 1902. A few human cases were reported between 1907 and 1928, but the town was free from plague for the next 60 years. A new outbreak occurred in 1991, followed by subsequent epidemics from 1995 to 1998 during which 1,702 suspected cases were reported [27]. In the capital Antananarivo, outbreaks of human plague were first recorded in 1921 [19]. After 58 years of silence, the disease reemerged in the city in 1979 with sporadic cases. Rodent surveillance initiated in the 1990s documented the replacement of *R. rattus* by *R. norvegicus* in the town (Table 1), favored by the construction of modern houses and sewage networks [3]. These changes were associated with a decrease in contact between humans and rat fleas due to the behavior of *R. norvegicus* [3]. Additionally, a lower susceptibility of *R. norvegicus* to plague also limited the risk of fleas leaving dead rodents in search of a new host, thus reducing human plague cases in Antananarivo [28].

### The Role of Endemic Fleas and Climate on Plague Epidemiology in Madagascar

Outside temperature may strongly affect flea abundance, thus affecting spatial and temporal distribution of the disease [29]. In Antananarivo and the surrounding highlands, plague cases are mostly reported during the warm rainy season from October to April. Conversely in Mahajanga, outbreaks of human plague occurred during the dry and cool season (from July to November). However, despite distinct plague seasons, the lowest temperature recorded in these two places during transmission is between 17° and 22°C [27], which can impact flea development. In the highlands, *S. fonquerniei* is exclusively found on rats caught outdoors and shows a clear seasonal cycle, thriving in the middle and at the end of the dry and cold season suggesting its role in initiating human plague epidemics [20]. This finding is supported by laboratory experiments suggesting that the development rate of flea larvae increases with temperatures below 30°C, and decreases above it. Furthermore, high temperatures with low humidity or temperatures below 9.3°C decrease the survival of the immature stages of *S. fonquerniei* [29]. In contrast, *X. cheopis*, which is mostly found on rats caught indoors, remains at relatively high abundance throughout the rainy season.

### Rats' Susceptibility to Plague in the Highlands of Madagascar

The susceptibility of rats to plague is undeniable. However, resistant *R. rattus* and *R. norvegicus* were reported in Antananarivo, which could explain the absence of epizootics and the maintenance of plague in the city [28]. Furthermore, whereas all rats in plague-free areas are sensitive to the disease, populations in plague-endemic areas are composed of sensitive and very resistant rats [28], [30]. The same was previously described for *R. pumilio* and *M. natalensis* in South Africa [31]. Yet the immune response to infection may differ even for the same species of rat within the same endemic area [32]. Dispersion of resistant rats with their fleas could support plague dissemination [14]. Ecology seems to support selection for resistance to plague as shown by genetic structure analysis of *R. rattus* populations in plague foci [33]. In Mandoto (peneplain area) no difference was found [33], whereas in Betafo (mountainous area) genetic differences were observed between rats from rice field populations compared to those from houses and sisal hedges [34]. This resistance seems to be passed on to offspring as also suggested for *M. natalensis* [31]. A 32-base pair deletion in the chemokine receptor 5 gene (CCR5) used by HIV-1 to enter cells has been proposed to confer resistance to HIV, smallpox, and plague infections [35]. Although experimental challenges with *Y. pestis* in normal and CCR5-Δ32 mice did not ascertain a protective role [35], [36], a unique substitution (H184R) in a region of the CCR5 gene was found to be more prevalent in resistant animals compared to susceptible ones and is more common in rats from plague foci than from plague-free areas [37]. Other genetic markers were investigated using an AFLP genome scan approach. Twenty-two loci have been identified that may be involved in the resistant phenotype of *R. rattus* found in the central highlands of Madagascar. Two loci were associated with plague infection outcome in experimentally challenged rats [38].

### Diversity of Reservoir Species

Although much less frequent and documented, sylvatic plague (Figure 3C) occurs in Malagasy primary forests where invasive *R. rattus* and endemic small mammals coexist and can sustain transmission through endemic fleas [25]. Human cases were reported among hunters and charcoal burners in these areas [3]. Both susceptible rodents and highly resistant insectivores live in these forests. Several endemic sylvatic small mammals such as shrews (Oryzorictinae subfamily) and tenrecs (Tenrecinae subfamily) were found infected by *Y. pestis* in sylvatic foci. They carry nonconventional vectors like *Paractenopsyllus* spp., *Tsaractenus* sp., and *Synopsyllus estradei* (unpublished data.). This mix of susceptible and resistant competent host species and potent vectors offers an explanation for epizootics and human plague cases. Deforestation also plays a major role in the dissemination of sylvatic plague to humans as seen in the Ikongo district after the introduction of *R. rattus* into this biotope. Endemic insectivores and hedgehogs in the forest were found seropositive in anti-F1 antibodies and substantiated an intense circulation of plague in this locality [39].

In the urban setting of Mahajanga, the Asian shrew, *Suncus murinus*, is most likely involved in plague transmission (Table 1). The abundance of *X. cheopis* on these shrews before the onset of human plague [3], the isolation of *Y. pestis* strains from their spleens [27], and their high seroprevalence after an epidemic period strongly suggests their involvement in the plague cycle. However, this hypothesis is questioned by the observation that *Y. pestis* strains isolated from *S. murinus* had different pulsotypes from those isolated from humans, rats, and fleas during the same outbreak [3].

### Plague Persistence in the Soil

During inter-epizootic periods, *Y. pestis* cannot be recovered from fleas, rodents, or any other host. Persistence of the bacteria in the soil was speculated in Iran and Madagascar [19], [40], [41]. Naive rodents may thus become infected by burrowing in contaminated soil (either via inhalation or ingestion), restarting a new cycle. Although the exact mechanism remains unclear, previous studies have demonstrated the survival of *Y. pestis* in soil for at least 24 days under natural conditions [42]. This was previously highlighted in 1963 by inoculation of guinea pigs with soil samples collected from burrows, containing remains of *Meriones vinogradovi* that had been dead from plague for 7–11 months [40]. This mode of persistence could explain inter-epizootic periods. However, the virulence of *Y. pestis* experimentally kept for one month in soil decreased considerably [19], and it was subsequently demonstrated that dry laterite highly inactivate *Y. pestis* [19]. Moreover, although *Y. pestis* may remain viable and virulent in soil, recent studies suggested that the transmission route by exposure of susceptible mice to *Y. pestis*–contaminated soil seems unlikely under natural conditions. Indeed, the infectious period was short-lived and the transmission efficiency is low [43].

### Human Behavior and Plague

Migration, poverty, and cultural practices can all have an impact on the incidence of human plague in Madagascar. A recent detailed SNP and MLVA analysis of *Y. pestis* strains evidenced multiple transfers of *Y. pestis* isolates between the highlands and Mahajanga harbor [44]. These transfers were most likely human-mediated, by transportation of goods containing infected rats or fleas by trucks or cars. In remote villages, people often prefer visiting traditional healers instead of health centers, thus delaying the implementation of an effective antibiotic treatment. Funeral ceremonies also favor the rapid spread of pneumonic plague [7], [20]. Indeed, a practice specific to Madagascar is to bury people in family burial vaults and to perform ritual corpse exhumations from time to time (Famadihana). Onsets of plague cases during these ceremonies have been observed, suggesting that handling of potentially plague-infected corpses may reactivate the disease. The Ministry of Health therefore recommended respecting a seven-year period between death and exhumation of a plague victim, and before any transfer of a corpse from one village to another. However, no study has been performed to determine the survival time of *Y. pestis* in corpses.

Poverty associated with overcrowded dwellings is another factor favoring rapid transmission and disease outbreaks in urban settings [3]. In villages, storage of crops within houses to prevent robbery attracts rats and their fleas [3], [7]. Agricultural activities, deforestation, and bushfires also promote spread of rats and dissemination of plague.

Finally, the discontinuation of plague surveillance since 2006 (due to financial shortages) has contributed to the reappearance of plague in the capital's suburbs six years after the last reported case. Two human cases were recently confirmed there outside the plague season, and *Y. pestis* was isolated from the spleen of *R. rattus*. The rat population in this area showed a higher than usual flea index, increasing the risk of *Y. pestis* transmission to humans and confirming that the disease is not under control, threatening the urban area of Antananarivo.

Effective plague prevention and control programs require up-to-date information on the incidence and the distribution of the disease. In Madagascar, plague surveillance (in humans and rodents) is a key priority for the Plague National Control Program (PNCP), established in 1993. The main objective of the PNCP is to reduce mortality due to plague and especially the mortality rate associated with the pneumonic form (<10% of notified cases) [7]. Surveillance is conducted by the Central Laboratory for Plague (CLP) of the Ministry of Health and the Plague Unit of the Institut Pasteur of Madagascar, which are the only facilities able to confirm plague in the country. Human surveillance is based on compulsory notification by health centers and on the biological confirmation of all suspected cases by the CLP. *Y. pestis* resistance to antibiotics currently used in plague treatment is also registered. The responsibility of health centers is the early detection of cases using the rapid diagnostic test at the patient's bedside to implement i) an appropriate treatment (streptomycin relayed by sulfonamide) for all suspected cases, ii) chemoprophylaxis (sulfonamide) for the contact population, and iii) the control of fleas [7]. The community is involved in passive surveillance of plague epizootics and rodent density.

## Conclusion

This review highlights the complexity of the epidemiology of plague in Madagascar and the effort made by past and present investigators to understand the reasons for the continuous presentation of human plague cases. Recent advances in various scientific fields have shown that the main host reservoir, the black rat populations of the highlands, are 1,000 times more resistant to plague than those from the coast. This is probably due to selective pressure. Adaptation of the plague bacillus to local ecological conditions may have also occurred, as suggested by the emergence and spread of new *Y. pestis* ribotypes in the most active foci of the highlands. The endemic flea *S. fonquerniei* may also play a significant role in the onset of the human plague season, whereas *X. cheopis* would be involved in sustaining disease transmission during several months thereafter. These various factors, along with human features, make the plague situation quite specific in Madagascar and reinforce the need for better surveillance. However, many questions still remain unanswered and represent future important challenges.

**Box 1. Key Learning Points**

1. Madagascar is among the top three countries that reported the most human plague cases during the past 15 years.
2. Plague occurs mainly as a rural disease, but also as an urban epidemic and a sylvatic transmission involving endemic rodents and fleas.
3. Plague is endemic in highlands above 800 metres of altitude with *R. rattus* as the main rodent reservoir and *X. cheopis* and the endemic flea *S. fonquerniei* as potential vectors.
4. Multidrug-resistant *Yersinia pestis* was first isolated in 1995 in Madagascar.
5. In less than a century, *R. rattus* has developed a strong resistance to the disease in endemic plague foci and this capability has a genetic basis.
6. Specific Malagasy traditions contribute to the rapid spread of pneumonic plague.

**Box 2. Key Papers in the Field**

1. Brygoo ER (1966) Epidemiologie de la peste à Madagascar. Arch Inst Pasteur Madagascar 35: 9–147.
2. Chanteau S (2006) Atlas de la peste à Madagascar. Paris: IRD Editions. 94 p.
3. Duplantier JM, Duchemin JB, Chanteau S, Carniel E (2005) From the recent lessons of the Malagasy foci towards a global understanding of the factors involved in plague reemergence. Vet Res 36: 437–453.
4. Rahalison L, Ranjalahy M, Duplantier JM, Duchemin JB, Ravelosaona J, et al. (2003) Susceptibility to plague of the rodents in Antananarivo, Madagascar. Adv Exp Med Biol 529: 439–442.
5. Migliani R, Chanteau S, Rahalison L, Ratsitorahina M, Boutin JP, et al. (2006) Epidemiological trends for human plague in Madagascar during the second half of the 20th century: a survey of 20,900 notified cases. Trop Med Int Health 11: 1228–1237.
